# A Word to Subscribers

**Published:** 1862-06

**Authors:** 


### A WORD TO SUBSCRIBERS.

The next number will complete the first volume of this journal. We have sent it regularly to the subscribers of the former *Buffalo Medical Journal*, and some others, presuming upon their desire to receive it.

We have now come to the trying point in publishing a medical journal, viz.: paying the printer; and we most earnestly invite all who receive it, and are willing to do so, to send us the *one dollar* for the first year's subscription. If this invitation is responded to, with promptness, we shall be able to liquidate all claims upon us, and start with our second volume with light heart and bright prospects. We hope the success of this journal is not to depend in any degree upon the amount of our private practice, but rather upon the favor and support of the profession, and that every physician who has made acquaintance with our young Journal, will be disposed to cultivate and extend it, giving to its pages contributions from their experience and observation, thus increasing its value and aiding its growth.

Our *necessity*, is sufficient excuse for calling attention to this matter of *money* at the present time. We must pay for the first volume or we cannot print the second.

We place the price of the *Journal* at only one dollar per year, just sufficient to pay expense of publication, and we expect that every physician will see how very important an item with us, that we receive that amount, receive it promptly, and receive it from all.

